# Multi-objective two-stage emergent blood transshipment-allocation in COVID-19 epidemic

**DOI:** 10.1007/s40747-023-00976-x

**Published:** 2023-02-24

**Authors:** Yufeng Zhou, Jiahao Cheng, Changzhi Wu, Kok Lay Teo

**Affiliations:** 1 Research Center for Economy of Upper Reaches of the Yangtze River, Chongqing Technology and Business University, Chongqing, 400067 China; 2 School of Business Administration, Chongqing Technology and Business University, Chongqing, 400067 China; 3 School of Management, Guangzhou University, Guangzhou, 510006 China; 4 School of Mathematics, Sunway University, 47500 Selangor Darul Ehsan, Malaysia

**Keywords:** Blood transshipment, COVID-19, Blood shortage, Blood supply chain, MOWOA

## Abstract

The problem of blood transshipment and allocation in the context of the COVID-19 epidemic has many new characteristics, such as two-stage, trans-regional, and multi-modal transportation. Considering these new characteristics, we propose a novel multi-objective optimization model for the two-stage emergent blood transshipment-allocation. The objectives considered are to optimize the quality of transshipped blood, the satisfaction of blood demand, and the overall cost including shortage penalty. An improved integer encoded hybrid multi-objective whale optimization algorithm (MOWOA) with greedy rules is then designed to solve the model. Numerical experiments demonstrate that our two-stage model is superior to one-stage optimization methods on all objectives. The degree of improvement ranges from 0.69 to 66.26%.

## Introduction

The outbreak of COVID-19 poses a serious threat to the safety of global blood supply chain [1]. Blood donations were canceled and the supply of blood was reduced suddenly in the United States in March 2020 [2]. Many countries are living with a 1- to 2-day stockpile inventory under the epidemics, whereas they used to be accustomed to a blood inventory of 1–2 weeks [3]. Blood registries in post-lockdown phase decreased by 35.13% compared to non-epidemic years in India [4]. In the first half of 2020, more than 30 large and medium-sized cities in Mainland China experienced a certain degree of blood stock shortage. The amount of whole blood collected in Beijing was less than $\frac{1}{3}$ of the average level in the same period of previous years. In Wuhan, the amount of whole blood collected was only about 10% in the same period in 2019. For this scenario, it is of great significance to integrate trans-regional blood transshipment, blood stocks replenishment in affected areas, and distribution of blood products to optimize the blood supply chain in affected areas.

Extensive research has been carried out on blood transshipment [5–7] and blood allocation [8, 9] under normal environment. Some researchers have also studied blood transshipment and allocation in natural disaster emergencies [10–13]. Different from previous studies, blood transshipment and allocation during COVID-19 are much more complicated. First, there are two distinct phases of blood transshipment and allocation during COVID-19. In the first stage of epidemic, the movement of people in the affected areas was strictly controlled. This has led to a sharp drop of blood donors in the local area, and as a result, the blood collection channels in the affected areas have been largely interrupted. In addition to the initial stockpile, replenishment of blood stocks can only be done through trans-regional transshipment. During the mitigation stage, the flow of people will partially recover. Then, blood banks in some low-risk areas can collect blood. In addition to replenishing through trans-regional transshipment, the blood inventory can also be replenished partially through local collection. The transshipment from other affected blood banks with low risk is also allowed. Second, the transshipment problem and the allocation problem under the epidemic affect each other. Obviously, integrating the two issues of trans-regional transshipment and optimal allocation is more conducive to the improvement of blood supply chain performance. Furthermore, the large-scale trans-regional transshipment of blood products involves the choice of multiple modes of transportation. The choice of different transportation modes will affect the quality of blood products received. Therefore, the problem should be defined as a two-stage trans-regional transshipment-allocation integrated decision-making problem with transportation mode selection.

Previous studies on expanding blood supply chain have usually pursued adequate supply and less obsolescence of blood products [14–16]. According to the characteristics of the epidemic blood supply chain, this paper expands the optimization objectives of the problem. First, this paper considers the effects of different transportation modes and initial freshness on the quality of blood products, and establishes an optimization objective for the best received blood product quality. Second, this paper establishes an optimal objective function for the satisfaction of blood products being allocated in affected areas, where the urgency of demand is taken into consideration. Third, the cost objective function established in this paper includes also the penalty cost of blood shortage. Furthermore, the scheduling process for blood products is a cold chain. In this paper, the cold chain cost of blood products is considered, which is rarely considered in the previous literature.

To sum up, this paper will consider the impact of different modes of transportation using two-stage multi-modal transport on blood product quality, full-process cold chain, and blood product distribution satisfaction during COVID-19. This problem is formulated as a multi-objective optimization problem to optimize the blood transshipment-allocation. The main differences between this paper and previous studies are summarized in Table 1.

**Table 1:** Features of relevant research

References	Multi-objective	Economic cost	Cold chain	Shortage	Multi stage	Blood quality	Satisfaction	Epidemic risk
[17]				$\checkmark$	$\checkmark$		$\checkmark$	$\checkmark$
[18]				$\checkmark$			$\checkmark$	
[19]		$\checkmark$		$\checkmark$		$\checkmark$		
[20]	$\checkmark$	$\checkmark$			$\checkmark$			
[21]		$\checkmark$		$\checkmark$	$\checkmark$			
[22]	$\checkmark$	$\checkmark$		$\checkmark$	$\checkmark$			
[6]	$\checkmark$			$\checkmark$		$\checkmark$	$\checkmark$	
[7]		$\checkmark$		$\checkmark$	$\checkmark$			
[23]		$\checkmark$		$\checkmark$		$\checkmark$		
[24]	$\checkmark$	$\checkmark$				$\checkmark$		
[25]		$\checkmark$		$\checkmark$			$\checkmark$	
[26]	$\checkmark$	$\checkmark$			$\checkmark$	$\checkmark$		
Our study	$\checkmark$	$\checkmark$	$\checkmark$	$\checkmark$	$\checkmark$	$\checkmark$	$\checkmark$	$\checkmark$

The contributions of this paper are as follows. (1) A new two-stage emergence blood transshipment-allocation during the COVID-19 epidemic is formulated as a multi-objective optimization problem. This problem stems from, but is not limited to, the blood supply chain industry. As mentioned in the previous subsection, the questions raised take into account many new characteristics of the epidemic, such as two-stage, trans-regional, and multi-modal transportation, the impact of different modes of transportation on the quality of blood products, the entire cold chain, and allocation satisfaction. (2) According to the characteristics of the model, a customized multi-objective whale optimization algorithm (MOWOA) with integer encoding and greedy rules is designed.

The rest of this paper is organized as follows. The relevant literature is reviewed in the section “Literature review”. The problem description and mathematical model are presented in the section “A two-stage transshipment-allocation model for emergency blood products”. The proposed hybrid MOWOA is discussed in the section “An improved hybrid MOWOA”. Computational experiments and a case study are conducted in the section “Case study and numerical analysis”. Conclusions and future work are presented in the section “Conclusions”.

## Literature review

This section will review relevant literature in the following three categories. Blood transshipment and allocation issues in normal environment, blood transshipment and allocation issues in emergencies, and blood supply chain management in COVID-19 epidemic.

### Blood transshipment and allocation problem in conventional environment

Due to the physiological characteristics of blood products, such as perishability, the transportation and allocation decision-making for blood products differ from general materials. In addition to economic cost, such studies often consider characteristics such as perishability and utilization. Reference [8] studies the Long Island blood distribution system. The system maximizes blood availability and utilization according to a programmed blood distribution system model and strategy. Reference [5] finds that the blood transfer between blood banks can effectively reduce the system cost. Reference [27] develops an integer programming model to solve the problem of blood distribution from a central blood bank to local hospitals. The model considers the different life cycles of blood distribution to reduce expiration. Reference [28] addresses a multiple-vehicle, multi-depot, multi-criteria allocation-routing problem for public and private health care systems. Reference [29] studies how to cost-effectively organize the delivery of blood products for Austrian hospitals. The approach being developed is based on integer programming and variable neighborhood search. Reference [30] studies the blood inventory control problem, taking into account blood group substitution and transshipment strategies. Reference [31] proposes the design and optimization of a blood distribution routes, and investigates the impact of inter-hospital compatible products’ substitution and transshipment on blood demand satisfaction. Reference [7] formulates a two-stage stochastic programming model for the proactive transshipment problem in the blood supply chain. The main objective is to establish an effective balance between the wastage and shortage. Reference [6] studies the dynamic decision-making of blood supply chain, and a bi-objective blood transshipment optimization model with the shortest transport time and the greatest freshness is established. According to the characteristics of the epidemic situation, this paper considers the blood transshipment, where the freshness of the transshipped blood products is taken into account. Furthermore, the paper also considers the influence of different modes of transportation on the quality of blood products.

### Blood transshipment and allocation problem in emergencies

Previous studies on emergency transshipment and allocation problem have mainly focused on earthquake [12]. Due to the unique physiological characteristics and safeguarding properties, emergency transshipment strategies for blood products are significantly different from other emergency supplies [18]. In [18], an age-based transshipment model for blood shortage is constructed, and two preferred selection methods for transshipping blood units are proposed. Reference [21] describes the uncertainty of post-disaster blood demand based on moment-based ambiguous set, and proposes a two-stage distributional robust optimization model to design the blood supply chain network. Reference [32] investigates the blood supply chain network design problem in emergency situations using a six-objective optimization framework. The problem is formulated as a mixed-integer linear programming model. Reference [33] studies robust location-allocation for emergency temporary blood supply after disasters. The focus of most blood allocation studies is on blood supply chain optimization, especially the problem of location-allocation or location-routing problem [34, 35].

In this paper, a multi-objective optimization model for integrated decision-making of blood transshipment and allocation is constructed based on the characteristics of epidemic.

### Blood supply chain management in the COVID-19 epidemic

The COVID-19 epidemic had led to global blood stock shortage [36]. Currently, there are little literatures on blood supply chain management in the COVID-19 epidemic. Blood supply chain management for the COVID-19 epidemic involves some new factors, such as virus variation, area risk, and the impact of human behavior on the transmission of the virus. Reference [37] introduces a plasma supply prediction model during the recovery phase of COVID-19 to help design a more efficient blood supply chain mechanism. Reference [38] proposes a robust multi-phase optimization method to model the blood supply network to ensure efficient blood collection. Reference [39] proposes a mechanism to coordinate activities to alleviate shortages during COVID-19 by providing a two-stage optimization tool. Reference [17] formulates the blood supply chain problem using a two-stage stochastic programming after the COVID-19 epidemic. Different from the literature mentioned above, this paper comprehensively considers some new characteristics of blood product scheduling in the context of COVID-19, such as the risk of affected areas, the impact of different modes of transportation on the quality of blood products, the whole cold chain, and allocation satisfaction. On this basis, a novel multi-objective optimization model for blood transshipment-allocation decision-making is proposed.

## A two-stage transshipment-allocation model for emergency blood products

### Problem description

**Fig. 1:**
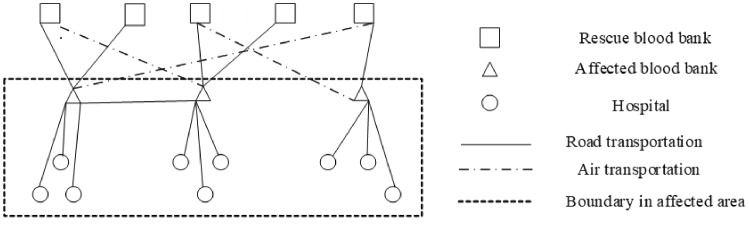
Two-stage transshipment-allocation diagram for emergency blood products

We suppose that the logistics network contains rescue blood banks, as shown in Fig. 1. There are multiple rescue blood banks and multiple affected blood banks. Each rescue blood bank serves multiple hospitals. Blood products are transshipped from the rescue banks to the affected banks, and each affected bank then allocates the blood products to its regional hospitals. During the first stage, blood collection from the affected areas is interrupted due to high risk. Blood bank replenishment can only be transshipped across regions. In the second stage, some blood banks in low-risk areas resume blood collection, and low-risk areas also allow the transshipment between affected blood banks.

There are multiple modes for blood transshipment, such as road transportation and air transportation. Different modes of transportation affect the quality of blood products. Therefore, in addition to the freshness of the transshipped blood, the quality of the received blood products will also depend on the choice of transportation modes. Furthermore, shortage penalty is included in the objectives to reduce shortage. In reality, blood shortage can have serious consequences, manifested in penalties such as delay in surgical procedure or additional costs for emergency blood collection. This setting has been widely adopted in blood supply chain management. Satisfactory distribution of blood products in affected areas during epidemic is also included as an optimization objective to ensure that urgent demands for blood products are met. Consequently, there are three objectives to be optimized in our model: (i) The quality of blood products being transshipped; (ii) the degree to which blood demands are met; and (iii) the total cost with out-of-stock penalties.

### Notations

(1) Sets

- *S*: Set of decision-making stages, $S=\left\{ s|s=1,2 \right\}$.
- *T*: Set of periods for each stage, $T=\left\{ t|t=1,2,...,{{t}_{\text {end}}} \right\}$.
- *I*: Set of rescue blood banks, $i\in I$.
- *J*: Set of affected blood banks, $j,n\in J$.
- *JH*: Set of hospitals served by blood bank *j*,$jh\in JH$. Here, the subscript *jh* represents hospital *h* in the area covered by blood bank *j*.
- *M*: Set of transportation modes,$m=\left\{ m|m=1,2 \right\}$, where $m=1$ denotes the road transportation, and $m=2$ denotes the air transportation.
- *P*: Set of blood products,$P=\left\{ p|p=wb,bp,rc \right\}$, where *wb*, *bp*, and *rc* represent whole blood, plasma, and red blood cell suspension, respectively
- (2) Parameters
- $d_{ij}^{m}$: Distance between location *i* and location *j* by using transportation mode.
- $v^{m}$: Velocity of transportation mode *m*.
- $\tau _{ij}^{m}$: Transportation time from *i* to *j* from using transportation mode *m*.
- ${{\tau }^{m}}$: The remaining time spent going from *i* to *j* from using transportation mode *m*. It includes the time spent from by loading and unloading goods and getting them to the airport.
- $C\text {a}{{\text {p}}^{m}}$: The capacity limit for the transportation mode *m*.
- $T{{U}_{s}}$: Transport time limit at stage *s*.
- $\Delta {{T}^{p}}$: Refrigeration temperature coefficient for blood product *p*.
- ${{c}_{s}}$: Unit penalty cost for shortage
- ${{c}_{m}}$: Unit transportation cost per unit of blood product from using transportation mode *m*.
- ${{c}_{e}}$: Unit blood refrigeration cost.
- ${{f}^{m}}$: The fixed cost of transportation per time from using transportation mode *m*.
- ${{\lambda }_{p}}$: Output coefficient for blood product *p*.
- $N(\mu _{jh}^{p},\sigma {{_{jh}^{p}}^{2}})$: Normal distribution of daily predicted demand with mean $\mu _{jh}^{p}$ and variance $\sigma {{_{jh}^{p}}^{2}}$ for blood product *p* in hospital *h* covered by blood bank *j*.
- $de_{jh,s}^{p}(t)$: Actual demand for blood product *p* in hospital *h* during period *t* at stage *s*.
- $\overline{B}_{i,s}^{p}$: Upper limit for the transshipment amount of blood product *p* from rescue blood bank *i* at stage *s*.
- $I_{j,0}^{p}$: Initial inventory level of blood product *p* in blood bank *j*.
- $I_{jh,0}^{p}$: Initial inventory level of blood product *p* in hospital *h* covered by blood bank *j*
- ${{Q}_{j,s}}(t)$: The amount of blood collected in blood bank *j* at stage *s*.
- $q_{j,s}^{p}(t)$: The amount of blood product *p* being prepared in blood bank *j* at stage *s* during period *t*.
- $r_{j,s}^{{}}$: If blood bank *j* is located in low-risk areas at stage *s*, it is 1; otherwise, it is 0.
- (3) Intermediate variables
- $I_{j,s}^{p}$: Inventory level of blood product *p* in blood bank *j* at the end of stage *s*.
- $I_{jh,s}^{p}$: The amount of inventory of blood product *p* that hospital *h* is being allocated by blood bank *j* at the end of stage *s*.
- $x_{j,s}^{p}$: The available amount of blood product *p* to be allocated in blood bank *j* at stage *s*.
- $R_{jh,s}^{p}$: The amount of shortage for blood product *p* in hospital *h* at stage *s*.
- (4) Decision variables
- $x_{ij,s}^{p}$: The amount of blood product *p* being transshipped from rescue bank *i* to affected bank *j* at stage *s*.
- $X_{jn,s}^{p}$: The amount blood product *p* being transshipped from affected bank *j* to affected bank *n* at stage *s*.
- $y_{ij,s}^{m}$: The used number of vehicles or airplanes from using transportation mode *m* to move from rescue bank *i* to affected bank *j* at stage *s*.
- $z_{jh,s}^{p}$: The allocated amount for blood product *p* from affected bank *j* to hospital *h* at stage *s*

### Objective functions

Based on the problem description mentioned above, the following three objective functions for the model are derived.

(1) The quality objective of the transshipped blood products.

Let ${{\theta }^{m}}$ be the deteriorating rate parameter for the transportation mode *m*. Expression (1) aims to maximizing the quality of blood products. Relevant research shows that the mode of transportation affects blood quality [40, 41]. Since air transportation has a greater impact on blood quality than road transportation over the same period, ${{\theta }^{2}}>{{\theta }^{1}}$. $f{{r}_{i,s}}$ is the blood quality at stage *s* from rescue bank *i*. $f{{r}_{i,s}}$ denotes the average ratio between the remaining shelf life to the total shelf life of the blood products transshipped from rescue bank *i*. The deterioration in the quality of blood products during short-term transportation can be measured as $\exp \left( -{{\theta }^{m}}(\tau _{ij}^{m}+\tau _{{}}^{m}) \right)$ [42, 43]. For the affected bank *j* at stage *s*, let ${{f}_{ij,s}}$ be the ratio of the received amount from the rescue bank *i* to the total received amount from all the rescue banks. Then, we have

1 $$\begin{aligned} {{f}_{ij,s}}=\sum \limits _{p\in P}{x_{ij,s}^{p}}/\sum \limits _{i\in I}{\sum \limits _{p\in P}{x_{ij,s}^{p}}},\quad \forall i,j,s. \end{aligned}$$

Now, the objective function of the quality of the transshipped blood products can be expressed as

2 $$\begin{aligned}{} & {} \max {{Z}_{1}}\left( s \right) =\sum \limits _{i\in I}\sum \limits _{j\in J}\nonumber \\{} & {} \quad {\sum \limits _{m\in M}{\exp \left( -{{\theta }^{m}}(\tau _{ij}^{m}+\tau _{{}}^{m}) \right) \cdot {{f}_{ij,s}}}}\cdot f{{r}_{i,s}}. \end{aligned}$$

(2) Objective function for the satisfaction of emergency blood allocation

The normalization of the time available for the inventory level of blood products at each stage can be calculated as

3 $$\begin{aligned}{} & {} \alpha _{jh,s}^{p}=\min \left\{ \frac{I_{jh,s-1}^{p}}{\mu _{jh,s}^{p} \cdot {{t}_{\text {end}}}},1 \right\} \quad \forall jh,s,p. \end{aligned}$$

Since blood demand varies from hospital to hospital, the predicted demand for each hospital is expressed dimensionless as

4 $$\begin{aligned}{} & {} \beta _{jh,s}^{p}=\frac{\mu _{jh,s}^{p}}{\max \mu _{jh,s}^{p}},\quad \forall jh,p,s. \end{aligned}$$

Because the shorter the time available for blood products in each hospital, the higher the urgency of the demand is. Thus, the demand urgency level $\eta _{jh,s}^{p}$ can be measured as expressed by

5 $$\begin{aligned}{} & {} \eta _{jh,s}^{p}=\sqrt{\left( 1-\alpha _{jh,s}^{p} \right) \beta _{jh,s}^{p}}, \quad \forall jh,s,p. \end{aligned}$$

If the stock of a hospital inventory is sufficient for current stage, i.e., $I_{jh,s-1}^{p}\ge \mu _{jh,s}^{p}\cdot {{t}_{end}}$, then the urgency level for the blood products is $\eta _{jh,s}^{p}=0$.

The actual demand satisfaction rate of the hospital can be calculated by the following formula:

6 $$\begin{aligned}{} & {} w_{jh,s}^{p}=\min \left\{ \frac{I_{jh,s-1}^{p}+z_{jh,s}^{p}}{\sum \nolimits _{t=1}^{T}{de_{jh,s}^{p}\left( t \right) }},1 \right\} ,\quad \forall jh,s,p. \end{aligned}$$

The satisfaction degree can be weighted by the urgency level $\eta _{jh,s}^{p}$, the actual demand satisfaction rate $w _{jh,s}^{p}$, and the demand proportion of each blood product $\frac{\mu _{jh,s}^{p}}{\sum \nolimits _{p\in P}{\mu _{jh,s}^{p}}}$. S0, at each stage, the satisfaction maximization objective in the context of demand urgency can be computed as follows:

7 $$\begin{aligned}{} & {} \max {{Z}_{2}}\left( s \right) =\sum \limits _{j\in J}\sum \limits _{h\in H}\sum \limits _{p\in P}{\eta _{jh,s}^{p}} \cdot w_{jh,s}^{p}\cdot \frac{\mu _{jh,s}^{p}}{\sum \nolimits _{p\in P}{\mu _{jh,s}^{p}}}. \end{aligned}$$

(3) Total cost objective including shortage penalty

The transportation cost between rescue banks and affected banks at each stage can be expressed as

8 $$\begin{aligned} {{c}_{1}}\left( s \right){} & {} =\sum \limits _{i\in I}{\sum \limits _{j\in J}{\sum \limits _{p\in P}{\sum \limits _{m\in M}{x_{ij,s}^{p}\cdot d_{ij}^{m}\cdot {{c}_{m}}}\cdot }}}y_{ij,s}^{m}\nonumber \\{} & {} \quad +\sum \limits _{i\in I}{\sum \limits _{j\in J}{\sum \nolimits _{m\in M}{{{f}^{m}}\cdot y_{ij,s}^{m}}}}, \end{aligned}$$

where the first item is the variable transportation cost, and the second item is the fixed transportation cost.

The transportation cost between affected banks at each stage can be expressed as

9 $$\begin{aligned} {{c}_{2}}\left( s \right){} & {} =\sum \limits _{j\in J}{\sum \limits _{n\in J}{\sum \limits _{p\in P}{X_{jn,s}^{p}\cdot d_{jn}^{{}}\cdot {{c}_{m}}\cdot }}} y_{jn,s}^{1}\nonumber \\{} & {} \quad +\sum \limits _{j\in J}{\sum \limits _{n\in J}{{{f}^{1}}\cdot y_{jn,s}^{1}}}. \end{aligned}$$

The blood refrigeration cost at each stage can be expressed as

10 $$\begin{aligned}{} & {} {{c}_{3}}\left( s \right) ={{c}_{e}}\sum \limits _{i\in I}\sum \limits _{j\in J} \sum \limits _{m\in M}\sum \limits _{p\in P}x_{ij,s}^{p}\cdot (\tau _{ij}^{m} +\tau _{{}}^{m})\cdot \Delta {{T}_{p}}. \end{aligned}$$

The penalty cost for shortage at each stage is given by

11 $$\begin{aligned} {{c}_{4}}\left( s \right) ={{c}_{s}}\sum \limits _{j\in J}{\sum \limits _{h\in H}{\sum \limits _{p\in P}{R_{jh,s}^{p}}}}. \end{aligned}$$

To sum up, the total cost objective function at each stage can be expressed as given below

12 $$\begin{aligned} \min {{Z}_{3}}\left( s \right) ={{c}_{1}}\left( s \right) +{{c}_{2}}\left( s \right) +{{c}_{3}}\left( s \right) +{{c}_{4}}\left( s \right) . \end{aligned}$$

### Mathematical formulation

A multi-objective optimization model for the two-stage transshipment-allocation decision-making of blood products in public health emergencies can now be formally stated as

13 $$\begin{aligned} \max {{Z}_{1}}&=\sum \limits _{s\in S}{{{Z}_{1}}\left( s \right) }\nonumber \\&=\sum \limits _{s\in S}\sum \limits _{i\in I}\sum \limits _{j\in J} \sum \limits _{m\in M}\exp \left( -{{\theta }^{m}}(\tau _{ij}^{m} +\tau _{{}}^{m}) \right) \nonumber \\&\quad \cdot {{f}_{ij,s}}\cdot f{{r}_{i,s}}, \end{aligned}$$

14 $$\begin{aligned}&\max {Z}_{2}=\sum \limits _{s\in S}{{{Z}_{2}}\left( s \right) }\nonumber \\&=\sum \limits _{s\in S}\sum \limits _{j\in J} {\sum \limits _{h\in H}{\sum \limits _{p\in P}{\eta _{jh,s}^{p}}\cdot }} w_{jh,s}^{p}\nonumber \\&\quad \cdot \frac{\mu _{jh,s}^{p}}{\sum \nolimits _{p\in P}{\mu _{jh,s}^{p}}}, \end{aligned}$$

15 $$\begin{aligned}&\min {Z}_{3}=\sum \limits _{s\in S}{{{Z}_{3}}\left( s \right) }\nonumber \\&=\sum \limits _{s\in S}\left[ {{c}_{1}}\left( s \right) +{{c}_{2}}\left( s \right) +{{c}_{3}}\left( s \right) +{{c}_{4}}{{\left( s \right) }} \right] , \end{aligned}$$

16 $$\begin{aligned}&\text {s.t. }q_{j,s}^{p}(t)\le {{\lambda }_{p}}\cdot {{Q}_{j,s}}(t)\cdot {{r}_{j,s}},\quad \forall j,s,t,p, \end{aligned}$$

17 $$\begin{aligned}&\tau _{ij}^{m}=\frac{di_{ij}^{m}}{{{v}^{m}}}\quad \forall i,j,m, \end{aligned}$$

18 $$\begin{aligned}&\tau _{ij}^{m}+\tau _{i}^{m}\le T{{U}_{s}}\quad \forall i,j,m,s, \end{aligned}$$

19 $$\begin{aligned}&x_{j,s}^{p}=\left\{ \begin{aligned}&\sum \limits _{i\in I}{x_{ij,s}^{p}+I_{j,0}^{p},s=1} \\&\sum \limits _{i\in I}{x_{ij,s}^{p}}+I_{j,s-1}^{p}\\ {}&+\sum \limits _{t=1}^{T}{q_{j,s}^{p}(t)},s=2 \\ \end{aligned} \right. \quad \forall j,p, \end{aligned}$$

20 $$\begin{aligned}&\sum \limits _{n\ne j}{X_{jn,s}^{p}}\cdot \sum \limits _{n\ne j}{X_{nj,s}^{p}}=0 \end{aligned}$$

21 $$\begin{aligned}&I_{j,s}^{p}=\left\{ \begin{aligned}&I_{j,s-1}^{p}+x_{j,s}^{p}-\sum \limits _{jh\in JH}{z_{jh,s}^{p}},s=1 \\&I_{j,s-1}^{p}+x_{j,s}^{p}+\sum \limits _{t=1}^{T}{q_{j,s}^{p}(t)}\\ {}&-\sum \limits _{jh\in JH}{z_{jh,s}^{p}},s=2 \\ \end{aligned} \right. \quad \forall j,p, \end{aligned}$$

22 $$\begin{aligned}&I_{jh,s}^{p}=\left[ I_{jh,s-1}^{p}+z_{jh,s}^{p}-\sum \limits _{t=1}^{T}{de_{jh,s}^{p}(t)}\right] ^{+}\quad \nonumber \\&\qquad \qquad \forall jh,s,p, \nonumber \\ \end{aligned}$$

23 $$\begin{aligned}&\sum \limits _{jh\in JH}{z_{jh,s}^{p}}\le x_{j,s}^{p}\quad \forall j,p,s \end{aligned}$$

24 $$\begin{aligned}&\sum \limits _{n\ne j}{X_{jn,s}^{p}}\le \left\{ \begin{aligned}&0,s=1 \\&(x_{j,s}^{p}-\sum \limits _{h\in H}{z_{jh,s}^{p}}){{r}_{j,s}},s=2 \\ \end{aligned} \right. \quad \forall j,p \end{aligned}$$

25 $$\begin{aligned}&R_{jh,s}^{p}=\left[ \sum \limits _{t=1}^{T}{de_{jh,s}^{p}(t)}-I_{jh,s-1}^{p}-z_{jh,s}^{p}\right] ^{+}\quad \nonumber \\&\qquad \qquad \forall jh,s,p, \nonumber \\ \end{aligned}$$

26 $$\begin{aligned}&\sum \limits _{j\in J}{x_{ij,s}^{p}\le \overline{B}_{i,s}^{p}\quad \forall i,s,p}, \end{aligned}$$

27 $$\begin{aligned}&\sum \limits _{p\in P}{x_{ij,s}^{p}} \le Ca{{p}_{m}}y_{ij,s}^{m}\quad \forall i,j,s, \end{aligned}$$

28 $$\begin{aligned}&{{r}_{j,s}}\in \left\{ 01 \right\} \quad \forall j,s, \end{aligned}$$

29 $$\begin{aligned}&x_{ij,s}^{p}X_{nj,s}^{p}z_{jh,s}^{p}\ge 0\quad \forall i,j,s,p,jh,m, \end{aligned}$$

30 $$\begin{aligned}&y_{ij,s}^{m}\in {{N}^{\text {*}}}\quad \forall i,j,s,m. \end{aligned}$$

The objective (13) is to maximize the quality of the transshipped blood. Equation (14) is the objective function to maximizing the satisfaction of blood demand. Equation (15) is the objective function to minimizing the total cost. Equation (16) is the restriction on the amounts of blood product collection, denoting that blood banks can only collect in low-risk areas. Equation (17) is the expression of the transportation time for different transportation modes. Equation (18) gives the limitations on the transport times. Equation (19) denotes the constraint on the allocation amounts available for blood products. Equation (20) is the equilibrium constraint for transshipped blood. Equation (21) is the expression of the inventory level of each blood bank at the end of each stage. Equation (22) denotes the expression of the inventory in each hospital at the end of each stage. Equation (23) is the constraint on the allocation amounts available for rescue blood banks. Equation (24) is the constraint on the transshipment amounts for affected blood banks. Equation (25) is the expression for the shortage in various hospitals. Equation (26) is the constraint on the transshipment amounts for rescue blood banks. Equation (27) is the constraint on the capacities for different transportation modes. Equations (28–30) are the constraints on the values of the variables.

### Estimation of the collected amount of blood products

In daily life, the number of donors visiting blood collection points *n* during each period *t* can be described as a Poisson distribution with the following distribution function [44]:

31 $$\begin{aligned} \Pr (n|\lambda t)=\frac{{{\lambda }^{n}}{{e}^{-\lambda t}}}{n!},n=0,1,2,...,\infty . \end{aligned}$$

After the outbreak of the epidemic, the arrival rate of donors has a large fluctuation. To simulate this, the average frequency of random events per unit time $\lambda$ is modeled as a Gamma distribution with the parameter $(\alpha ,\beta )$ given as follows:

32 $$\begin{aligned}{} & {} g(\lambda )=\frac{1}{\Gamma (\alpha ){{\beta }^{\alpha }}} {{\lambda }^{\left( \alpha -1 \right) }}{{e}^{-\lambda /\beta }},0\le \lambda <\infty . \end{aligned}$$

Different values of $(\alpha ,\beta )$ are being used to reflect the variation of donors visiting blood collection locations during epidemic. Suppose that the probability of each visitor meeting the conditions for blood collection is *p*. Then, the probability distribution of the number $\overline{n}$ of eligible donors among the number *n* of people who visit blood collection locations in each cycle after the outbreak of epidemic can be expressed as

33 $$\begin{aligned}{} & {} \Pr (\overline{n}|n)=C_{n}^{\overline{n}}{{(1-p)}^{(n-\overline{n})}} {{p}^{\overline{n}}},\overline{n}=0,1,2,...,n. \end{aligned}$$

Therefore, the probability distribution of eligible donors within a period is given by

34 $$\begin{aligned} \begin{aligned} \Pr (\overline{n})&=\int _{\lambda =0}^{\infty } {\sum \limits _{n=0}^{\infty }{\Pr (\overline{n}|n)\Pr (n|\lambda )}}\\ g(\lambda )d\lambda&=C_{\overline{n}}^{\overline{n}+\alpha -1} {{\left( \frac{\beta tp}{1+\beta tp}\right) }^{\overline{n}}} {{\left( \frac{1}{1+\beta tp}\right) }^{\alpha }},\\ {}&n=0,1,2,...,\infty ;\overline{n}=0,1,2,...,n. \end{aligned} \end{aligned}$$

Thus, the expected value of $\overline{n}$ is

35 $$\begin{aligned} E(\overline{n})=\alpha \beta tp. \end{aligned}$$

Supposing that the average blood donation volume of each donor is *q*, then the upper limit of blood collection in each region during a period is

36 $$\begin{aligned} Q_{j,s}^{t}={{\alpha }_{j,s}}{{\beta }_{j,s}}tpq. \end{aligned}$$

## An improved hybrid MOWOA

The model proposed in this paper is a multi-objective mixed-integer nonlinear programming model. This problem is an NP-hard problem, which is difficult to solve accurately with branch-and-bound algorithm, or operational research software such as CPLEX and GUROBI. Meta-heuristic algorithms are found to have better applicability to solve such problems.

Methods for solving multi-objective problems can be roughly divided into two categories. For the first category, the multi-objective problem is transformed into a single objective problem using linear weighting or the TOPSIS method. For the second category, the task is to obtain the Pareto frontier solution set. Decision-makers can select a satisfactory solution from the Pareto solution set according to the actual situation.

Whale Optimization Algorithm (WOA) was proposed in 2016 and has received widespread attention due to its fast convergence speed and few parameters [45]. The MOWOA was introduced in [46] based on the traditional WOA. It has been widely used to solve various multi-objective combinatorial optimization problems. Based on the characteristics of the proposed model, an improved hybrid MOWOA with greedy rules is designed in this paper. The performance of our proposed hybrid MOWOA is compared with that of NSGA-II, MODE, and MOGWO.

The reason why MODE and NSGA-II are chosen for comparison is that these two algorithms are the most classic multi-objective evolutionary algorithms and are widely used. MOGWO was chosen, because it is a relatively new optimization algorithm that performs well in some cases [47].

### Whale position encoding

**Fig. 2:**
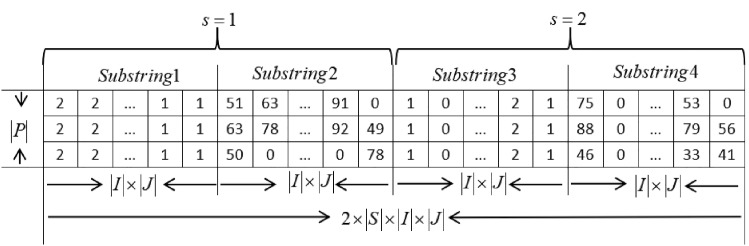
Encoding schematic for a chromosome

The encoding of a whale position is designed as a matrix. Each row of the matrix represents a blood product. The row is used to encoding the transportation mode and transshipment amount of a blood product. Each row has four substrings. The first two substrings are used to encode variables of stage 1, and the last two substrings are used to encode variables of stage 2. Each of the four substrings contains $\left| I \right|$ position segments. Each position segment represents a rescue blood bank. Each position segment has $\left| J \right|$ bits. Therefore, each substring has $\left| I \right| \times \left| J \right|$ positions. A complete whale position is a matrix with size $\left| P \right| \times \left[ 2\times S\times \left( \left| I \right| \times \left| J \right| \right) \right]$, as shown in Fig. 2.

**Fig. 3:**
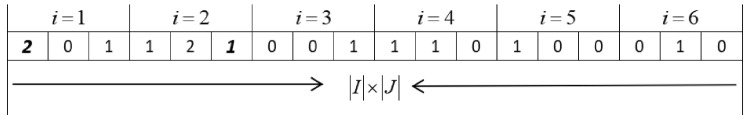
Encoding schematic for substring 1

In substring 1, each bit is randomly generated between $\{0,1,2\}$. Different values of bits indicate different transportation modes. 0 means no transshipment between the two banks. 1 and 2 indicate, respectively, the road and air transportation modes, that is, $m=1$ or $m=2$. Suppose there are six rescue blood banks and three affected blood banks. As shown in Fig. 3, the number of bits in substring 1 is 18. The first bit means that affected bank 1 is transferred from rescue bank 1, and the selected mode is air transportation, i.e., $m=2$. The sixth bit means that affected bank 3 is transferred from rescue bank 2, and the selected mode is road transportation, i.e., $m=1$, and so on.

**Fig. 4:**
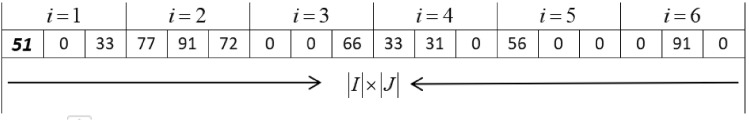
Encoding schematic for substring 2

Substring 2 is used to encode transshipment amount $x_{ij,s}^{p}$. Each bit of the initial population is a positive integer randomly generated among $\left[ 0,\overline{B}_{i,s}^{p} \right]$. Assuming that substring 2 shown in Fig. 4 is at row *p* and stage *s* of the chromosomal encoding matrix. The first bit means that transshipment amount for blood product *p* from rescue bank 1 to affected bank 1 at stage *s* is 51*U*, and so on for other bits.

Substring 3 and substring 4 are the encoding for stage 2. Substring 3 is encoded in the same way as substring 1, and the encoding of substring 4 is the same as substring 2.

The encoding of other blood products, that is the other rows of the matrix, can also be encoded similarly as substring 1 to substring 4 depicted above.

After the initial encoding is generated, the encoding needs to be moderately improved, and the improvement rules are as follows.

1. In the same row of a whale position, if the bit of substring 1 is 0 and substring 2 is >0 for the corresponding bit position of substring 1 and substring 2, the bit of substring 2 should be modified to 0. This means that there is no transshipment between the two blood banks, and the transshipment amount should be modified to 0. Encoding of substring 3 and substring 4 in the same row should be improved in the same way.
2. If the bits with the same serial number of substring 1 in different rows of whale positions are different, change the value of bits in all rows to the maximum value of these bits. This operation can unify the transportation mode between the blood banks into one mode, and can also make different blood products to transship together. The encoding of substring 2 in different rows should also be modified in the same way.

$x_{j,s}^{p}$ can be calculated based on $x_{ij,s}^{p}$. $z_{jh,s}^{p}$ can be obtained using greedy heuristic rule as detailed in Step 3.3 to be given below.

###  Algorithm procedure

**Step 1 Algorithm initialization.** Set the number of whale population as *L* and the maximum iterations as $T_{\max }$.

**Step 2 Initialization of whale positions.** Generate *N* whales randomly according to the encoding rules in “Whale position encoding”, the position $\vec {X}_{i}(i=1,2,3, \ldots l)$ of each whale is an initial solution. Parameters $\vec {a}, \vec {A}, \vec {C}, p$ should be initialized, too, where $\vec {A}, \vec {C}$ are coefficient vectors, which are used to control the whale’s swimming mode. The calculation method is shown in Eqs. (37)–(39)

37 $$\begin{aligned}{} & {} \vec {A}=2 \vec {a} \cdot \vec {r}-\vec {a} \end{aligned}$$

38 $$\begin{aligned}{} & {} \vec {C}=2 \vec {r} \end{aligned}$$

39 $$\begin{aligned}{} & {} \vec {a}=2-2 t / T_{\max }. \end{aligned}$$

Here, $\vec {a}$ is the control parameter, $\vec {r}$ is a random vector between [0,1], and *t* is the current iterations.

**Step 3 Fitness value calculation.** The steps are as follows.

**Step 3.1** For individual *l* and stage 1, transport mode m and transshipment amount $x_{ij,s}^{p}$ can be obtained according to the decoding method in “Whale position encoding” .

**Step 3.2** Calculate $\sum \nolimits _{p\in P}{x_{ij,s}^{p}},\forall i,j,s=1$. Then, the variables $y_{ij,s}^{m},\forall i,j,m,s=1$ can be obtained from constraint (27).

**Step 3.3** Calculate the allocated amounts $z_{jh,s}^{p},\forall jh,p,s=1$ based on the greedy rule. First, calculate the level of urgency of demands $\eta _{jh,s}^{p},\forall jh,p,s=1$ according to Eqs. (3)–(5). Then, select the maximal $\eta _{jh,s}^{p},\forall jh,p,s=1$ and hospital *jh*. The allocated amount for this hospital can be calculated by $z_{jh,s}^{p}=\sum \nolimits _{t=1}^{T}{de_{jh,s}^{p}\left( t \right) }-I_{jh,s-1}^{p},\forall jh,p,s=1$. Update $x_{j,s}^{p}=x_{j,s}^{p}\text {-}z_{jh,s}^{p},\forall j,p,s=1$. Then, allocate the demand of the hospital with the second largest $\eta _{jh,s}^{p},\forall jh,p,s=1$. The same process is carried out for the other hospitals until all blood banks have allocated all blood products to hospitals.

**Step 3.4** Update $I_{j,s}^{p},\forall j,p,s=1$; $I_{jh,s}^p,\forall jh,p,s=1,R_{jh,s}^{p},\forall jh,p,s=1$ according to the actual demands $de_{jh,s}^{p}(t),\forall jh,p,s=1$.

**Step 3.5** For stage 2, if $r_{j,s}^{{}}=1,\forall j,s=2$, update $q_{j,s}^{p}(t)$ according to constraint (13). The calculations of $x_{j,s}^{p},\forall j,p,s=2$, and then, $\sum \nolimits _{p\in P}{x_{ij,s}^{p}},\forall i,j,s=2$ are carried out similar to Steps 3.2 and 3.3.

**Step 3.6** For the two affected banks *j*1 and *j*2, if $\forall r_{j1,2}^{{}}=1, r_{j2,2}^{{}}=1$, and $I_{j1,2}^{p}>0,\forall p$, $\sum \nolimits _{jh\in JH}{R_{jh,2}^{p}}>0,\forall p$, then the blood products are transshipped from *j*1 to *j*2, and $X_{j1j2,2}^{p}=\min \{I_{1,2}^{p},\sum \nolimits _{jh\in JH}{R_{jh,2}^{p}}\},\forall p$.

**Step 3.7** Calculate the fitness value of individual *l*. Let $Fit(l)=\{{{f}_{1}},{{f}_{2}},{{f}_{3}}\}$, where ${{f}_{1}},{{f}_{2}},{{f}_{3}}$ are, respectively, the values of the 3 objective functions.

**Step 3.8** Calculate the fitness values of all individuals as in Steps 3.1 to 3.7.

**Step 4 Non-dominant sorting**. For convenience of non-dominant sorting, let ${{f}_{1}}=-{{f}_{1}},{{f}_{2}}=-{{f}_{2}}$. Then, it performs a non-dominant sorting for all individuals as done using the traditional methods.

**Step 5 Crowding calculation**. The crowding distance of individuals of the same non-dominant rank $F\{i\}$ can be calculated according to the fitness value as given below

40 $$\begin{aligned}{} & {} di({{l}_{1}},{{l}_{2}})=\sqrt{\sum \limits _{i=1}^{3} {{{\left( \frac{{{f}_{i}}({{l}_{1}})-{{f}_{i}}({{l}_{2}})}{f_{i}^{\max }-f_{i}^{\min }} \right) }^{2}}}}. \end{aligned}$$

**Fig. 5:**
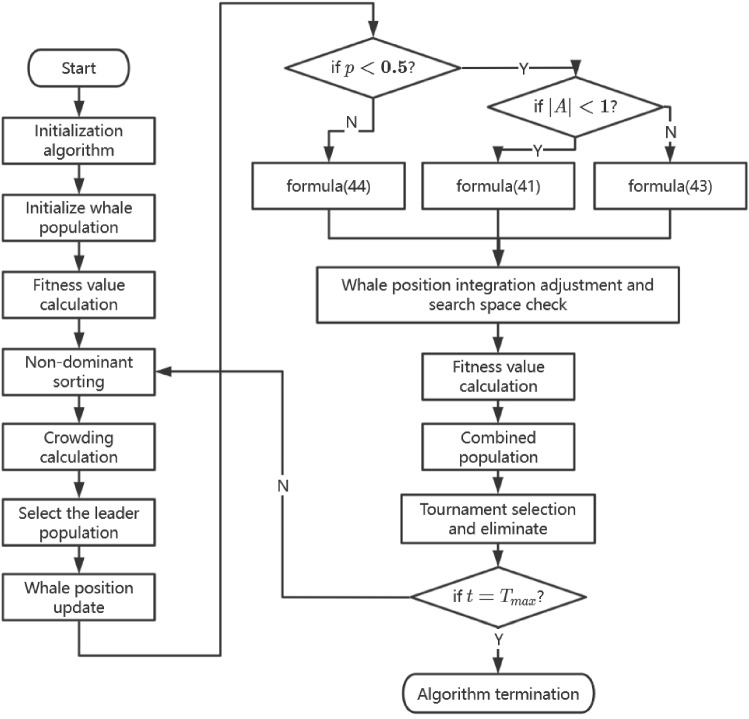
Algorithm flowchart

**Step 6 Leading population selection.** Whales in the first non-dominant tier should be selected as leaders to form the leading population.

**Step 7 Whale position updating.** Update the position of all whales as follows.

1. If $p<0.5$ and $|\vec {A}|<1$, the whale makes a movement to surround the prey, and the position of the whale can be updated according to the following formula:
  41 $$\begin{aligned} \vec {X}(t+1)=\vec {X}^{*}(t)-\vec {A} \cdot \vec {D}_{1}. \end{aligned}$$
  Here, $\vec {X}^{*}$ is the position vector of the randomly selected whale in the leading population, which is set as position of the target prey. $\vec {D}_{1}$ is the distance between the individual whale and the prey, and the calculation method is as follows:
  42 $$\begin{aligned} \vec {D}_{1}=\left| \vec {C} \cdot \vec {X}^{*}(t)-\vec {X}^{*}(t)\right| . \end{aligned}$$
2. If $p<0.5$ and $|\vec {A}|>1$, when the whale searches for prey, it no longer updates its position according to the leading whale, but randomly selects a whale position in the current population to update its position. The diversity of the population is maintained while seeking the optimal solution. The position of the whale is updated according to the following formula:
  43 $$\begin{aligned} \vec {X}(t+1)=\vec {X}_{\text{ rand } }-\vec {A} \cdot \vec {D}_{\text{ rand } }. \end{aligned}$$
  Here, $\vec {X}_{\text{ rand } }$ represents the randomly selected whale position vector, and $\vec {D}_{\text{ rand } }$ can be calculated in the same way as Eq. (42).
3. If $p \ge 0.5$, the whale makes a spiral position updating action. The spiral equation is used to simulate this process. The whale position is updated according to the following formula:
  44 $$\begin{aligned} \vec {X}(t+1)=\left| \vec {X}^{*}(t)-\vec {X}(t)\right| \cdot e^{b v} \cdot \cos (2 \pi v)+\vec {X}^{*}(t). \nonumber \\ \end{aligned}$$
  Here, *b* is a constant used to define the logarithmic spiral shape, and *v* is a random number between [– 1,1].

**Table 2:** Transportation distance between rescue banks and affected banks (km)

Distance (air/road)		Rescue banks								
		CQ	CD	CS	GY	NJ	NC	HF	ZZ	XA
Affected banks	WH	754/944	979/1156	297/329	865/1044	457/536	262/341	310/378	467/510	653/739
	YC	471/581	694/834	319/401	636/859	728/842	495/636	575/688	503/625	453/683
	XY	–/802	–/976	–/524	–/1029	–/768	–/647	–/613	–/392	–/510
	HG	–/946	–/1236	–/386	–/1100	–/512	–/290	–/357	–/576	–/849
	JZ	–/667	–/933	–/330	–/825	–/749	–/537	–/594	–/586	–/704

*CQ* Chongqing, *CD* Chengdu, *CS* Changsha, *NJ* Nanjing, *NC* Nanchang, *HF* Hefei, *ZZ* Zhengzhou, *XA* Xi$\prime$an, *WH* Wuhan, *YC* Yichang, *XY* Xiangyang, *HG* Huanggang, *JZ* Jinzhou

**Table 3:** Upper limits of the transshipment amounts for blood products from rescue blood banks

Stage1/ Stage 2	CQ	CD	CS	GY	NJ	NC	HF	ZZ	XA
*wb*(U)	809/443	839/460	639/359	822/450	791/434	648/364	769/423	730/406	799/401
*bp*(U)	3236/1770	3357/1839	2557/1432	3287/1804	3165/1743	2582/1451	3076/1698	2900/1610	3160/1732
*rc*(U)	4046/2213	4197/2299	3197/1799	4109/2255	3957/2179	3228/1814	3846/2123	3625/2013	3950/2075
Quality	0.95/0.98	0.96/0.97	0.93/0.96	0.90/0.95	0.91/0.92	0.92/0.97	0.95/0.92	0.97/0.94	0.91/0.93

**Step 8 Whale position adjustment and search space check.** Round down the whale position and check if it is outside the search space. Here, the transport modes of substring 1 and substring 3 are directly rounded down. The transshipment amount of substring 2 and substring 4 are updated and rounded down as follows:

1. If $\sum \nolimits _{j \in J}{x_{i j, s}^{p}}\le \bar{B}_{i, s}^{p}, \forall i, s, p$, $x_{i j, s}^{p}$ are rounded down directly.
2. If $\sum \nolimits _{j \in J}{x_{i j, s}^{p}} > \bar{B}_{i, s}^{p}, \forall i, s, p$, $x_{i j, s}^{p}$, are updated according to (45) and then rounded down
  45 $$\begin{aligned}{} & {} x_{i j, s}^{p}=\left\lceil \frac{x_{i j, s}^{p}}{\sum \limits _{j\in J}{x_{i j, s}^{p}}} \cdot \bar{B}_{i, s}^{p}\right\rceil , \quad \forall i, s, p. \end{aligned}$$

**Step 9 Fitness value calculation.** Calculate the fitness value of the new whale population as in Step 3.

**Step 10 Population merging.** The new population is merged with the original population, and the Pareto level and crowding degree distance of the population are calculated according to Step 4 and Step 5.

**Step 11 Tournament selection.**The non-dominant rank of the initial population is compared, and then, the crowding distance is compared. The individuals with larger non-dominant rank and smaller crowding distance are selected to enter the next step.

**Step 12 Individual elimination and selection.** Select the former *L* individuals to enter the next generation according to the method of Step 11 and eliminate the other individuals to maintain the same population size.

**Step 13 Algorithm termination.** If $t=T_{\max }$, then the algorithm terminates. Otherwise, return to Step 4.

The algorithm flowchart is given in Fig. 5.

**Table 4:** Average daily blood consumption in Wuhan hospitals

Product/ Hospital	1	2	3	4	5	6	7	8	9	10
*wb*(U)	14	12	13	12	12	14	14	12	14	12
*bp*(U)	56	48	54	53	50	55	55	50	55	53
*rc*(U)	70	60	67	66	63	69	69	63	69	67
Product/ Hospital	11	12	13	14	15	16	17	18	19	20
*wb*(U)	15	15	14	15	13	14	14	12	14	13
*bp*(U)	59	60	56	59	52	56	55	49	55	53
*rc*(U)	73	73	71	73	66	70	69	62	68	67

**Table 5:** Initial inventory levels and reserve amounts for different affected banks

Product/bank	WH		YC		XY		HG		JZ	
	$I_{j,0}^{p}$	$q_{j,s}^{p}(t)$	$I_{j,0}^{p}$	$q_{j,s}^{p}(t)$	$I_{j,0}^{p}$	$q_{j,s}^{p}(t)$	$I_{j,0}^{p}$	$q_{j,s}^{p}(t)$	$I_{j,0}^{p}$	$q_{j,s}^{p}(t)$
*wb*(U)	776	–	664	40	579	40	463	–	558	40
*bp*(U)	3103	–	2656	160	2313	160	1852	–	2232	160
*rc*(U)	3879	–	3320	200	2891	200	2315	–	2789	200
${{r}_{j,s}}$	0		1		1		0		1	

**Fig. 6:**
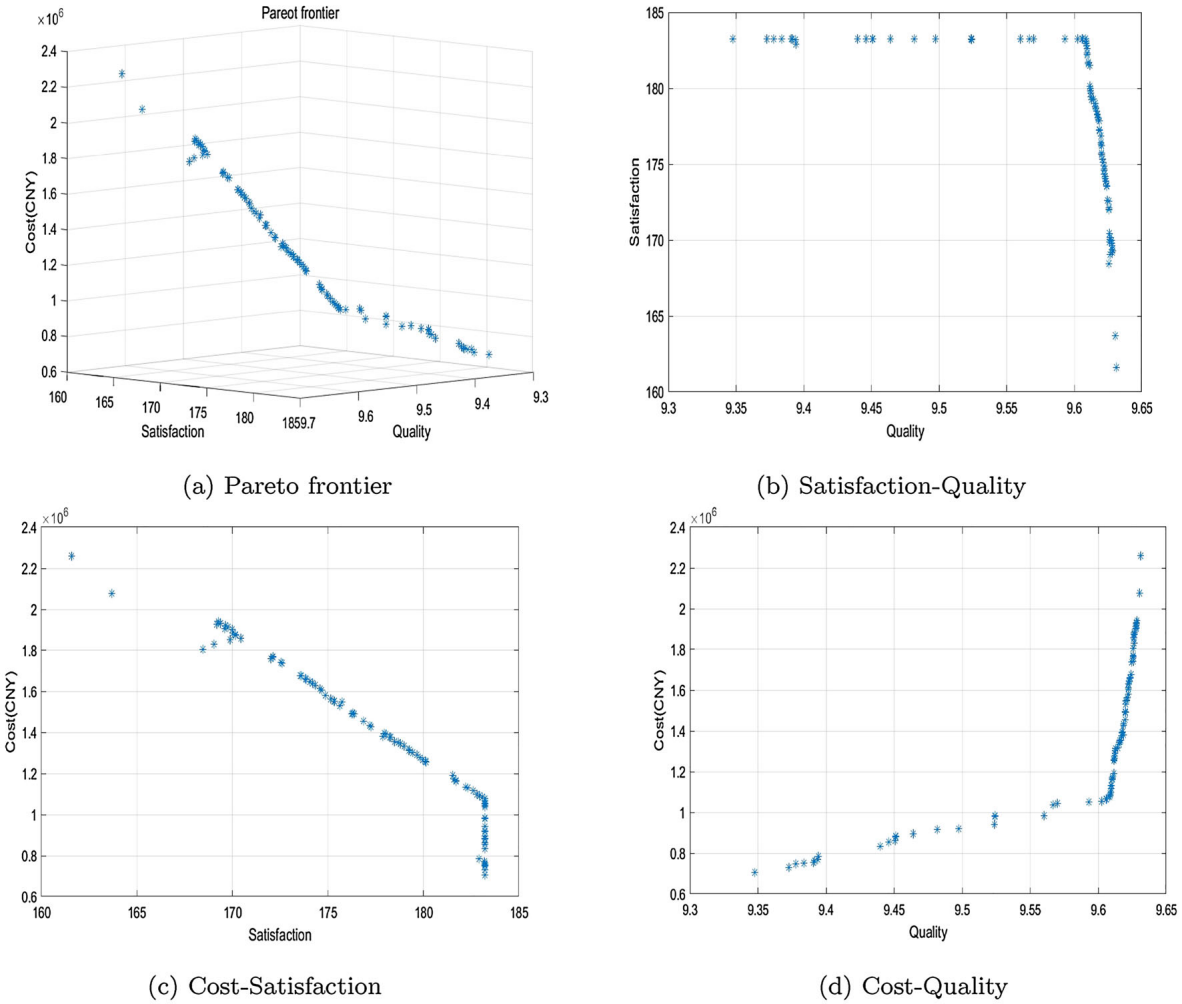
Solution results

## Case study and numerical analysis

### Case description and data

In this section, we construct a numerical example based on the epidemic in Hubei Province, China to evaluate our proposed model. On August 5th 2021, Wuhan and some cities in Hubei province experienced second wave of outbreaks since January 2020. 63 residential areas in Wuhan were under lockdown for quarantine management. The Government conducted a nationwide blood transshipment to Hubei province. The rescue blood banks are blood centers in Chongqing, Chengdu and other 9 cities, labeled as $i=1,2,...,9$. Wuhan, Yichang, Xiangyang, Huanggang, and Jinzhou are the five major cities that received transshipped blood. Blood banks in these cities are affected banks. The air and land transportation distances between different blood banks are measured by actual routes and highway kilometers, as shown in Table 2.

The upper limits of the transshipment volumes of blood products from rescue blood banks are shown in Table 3. The daily blood demands in hospitals are given based on historical data. Table 4 shows the average daily demand data of 20 hospitals in Wuhan. The initial inventory levels of blood products in affected banks are shown in Table 5. The amount of blood collected is calculated according to Equations (31)–(36), and the relevant parameters are set as $\alpha =20,\beta =20, q=1.5, p=2/3$. The preparation volume of blood products is calculated based on the collected volume, as shown in Table 5.

In the first week after the outbreak of epidemic, the five cities were in high-risk state. The epidemic prevention and control were strict, the movement of people was restricted, and the amount of blood collected dropped rapidly. At this stage, blood consumption mainly depends on the stocks of affected blood banks and the transshipment from rescue banks. Here, all affected banks are located in high-risk areas, and ${{r}_{j,s}}=0,\forall j,s=1$. After a week of control, the epidemic in some cities has not spread, and blood collection and transfer between affected areas have begun. Therefore, stage 1 is set as from the 1st to the 7th day after the outbreak, and stage 2 is set as from the 8th to the 14th day after the outbreak.

The other parameters of the model are set as follows. ${{v}^{1}}=100$ km/h, ${{v}^{2}}=800$ km/h, ${{\tau }^{1}}=1$ h, ${{\tau }^{2}}=2$ h, $Ca{{p}^{1}}=2000$U, $Ca{{p}^{2}}=10,000$U, $T{{U}_{1}}=12$ h, $T{{U}_{2}}=24$ h, ${{\theta }^{1}}=0.01$, ${{\theta }^{2}}=0.1$, ${{c}_{s}}=100$CNY, ${{c}_{1}}=0.01$CNY, ${{c}_{2}}=0.02$CNY, ${{c}_{e}}=0.1$CNY, ${{f}_{1}}=500$CNY, ${{f}_{2}}=2000$CNY. The parameters of MOWOA are set as follows. The whale population $L=100$, and the maximum iterations $T_{\max }=400$.

### Results

MatlabR2019a is used as programming environment. The running platform is a personal computer with AMD Ryzen 7 5800 H, Radeon Graphics CPU@3.2GHz, 16GB ram, 64-bit Windows 11 system. The running time is 131 s. When the algorithm terminates, all the solutions converge to the Pareto front surface, and there are 100 distinct Pareto solutions. The distribution of solutions is shown in Fig. 6a. Figure 6b is the surface fitted by Pareto solutions. Figure 6c and d shows distribution diagrams of solutions between different objectives. Some typical Pareto solutions are shown in Table 6.

From Fig. 6 and Table 6, it can be concluded that the total cost objective is opposite of the freshness objective. There is no conflict between satisfaction and cost, because the shortage penalty is considered. As shown in the right end of Fig. 6c, when satisfaction levels rise to a high level, optimizing the transshipment scheme can significantly reduce the total cost. Excessive pursuit of the quality of transshipment blood will concentrate the selection of rescue blood banks in cities close to the affected areas, resulting in a large shortage cost.

### Algorithm comparison

**Table 6:** Some typical Pareto solutions

No.	Objective value	Stage	${{x}_{ij,s}}$(U)									Shortage (U)
1	${{z}_{1}}=9.348$ ${{z}_{2}}=183.229$ ${{z}_{3}}=706799$	1	1821	4318	330	4538	3320	772	241	636	1492	0
			3871	1168	329	1955	1900	314	674	80	1199	
			0	0	0	0	0	0	217	5534	4781	
			0	0	0	551	0	4947	6137	0	0	
			749	0	5729	1162	0	378	419	865	0	
		2	3604	1892	511	1262	1950	268	668	541	2430	
			0	2440	554	3014	1326	194	361	571	1398	
			328	0	0	182	0	241	496	2655	70	
			0	0	132	0	60	2561	501	0	111	
			0	174	1992	0	25	340	2200	0	317	
2	${{z}_{1}}=9.631$ ${{z}_{2}}=161.560$ ${{z}_{3}}=2259484$	1	2723	2854	169	3003	5537	712	670	521	6675	11,590
			5351	5505	216	5177	2372	518	741	176	1223	
			0	0	0	0	0	0	0	6550	0	
			0	0	0	36	0	5186	0	0	0	
			0	0	6006	0	0	37	0	0	0	
		2	1781	1217	49	2038	2819	417	2766	191	1420	
			2615	3359	104	2450	1517	278	1455	168	2908	
			0	0	0	0	0	0	0	3644	0	
			0	0	0	0	0	2910	0	0	0	
			27	0	3417	0	0	0	0	0	0	
3	${{z}_{1}}=9.619$ ${{z}_{2}}=176.849$ ${{z}_{3}}=1454537$	1	2664	2695	117	2832	5577	259	522	396	1034	3728
			5397	5695	144	5376	2334	183	314	154	640	
			0	0	0	0	0	0	0	6689	6215	
			11	0	0	0	0	5086	6852	0	0	
			17	0	6128	0	0	926	0	0	0	
		2	1740	1461	85	2046	2768	122	2677	132	1436	
			2684	3111	68	2442	1562	97	1547	89	2872	
			0	0	0	0	0	0	0	3777	19	
			0	0	0	0	0	3387	0	0	0	
			0	0	3418	0	0	0	0	0	0	

For ${{x}_{ij,s}}$, columns represent affected blood banks and rows represent rescue blood banks

To test the performance of the proposed improved MOWOA in this paper, it is compared with NSGA-II [48], MODE [49], and MOGWO [47]. Nine numerical examples are designed for comparison. Numerical Example 1 is the case constructed above. The other eight examples are randomly generated based on Example 1 with different sizes. The maximum iterations of the three algorithms are all 400. The population size (individual number/gray wolf population) is 100. The three comparison algorithms all use their original approaches, but to solve our proposed model, they all use the same encoding and decoding rules proposed in this paper.

We use four indicators to evaluate the performance of these algorithms. Tiers represent the final number of Pareto levels. If it is not 1, there is no convergence. CPU time is used to measure the computational efficiency of these algorithms. Different Pareto solutions represent the diversity of solutions. HV is used to evaluate the convergence and distribution of solutions simultaneously [50]. HV is defined as follows:

46 $$\begin{aligned} H V\left( S, z^{\text {ref}}\right) =\text {volume}\left( \bigcup _{i=1}^{|S|} c^{i}\right) . \end{aligned}$$

Here, $z^{\text {ref}}$ is a pre-set reference point, and |*S*| is the number of non-dominant solution sets. The volume $volume \left( c^{i}\right)$ of the hypercube formed by a non-dominated solution $x^{i}$ and a reference point as diagonal lines is calculated as follows:

47 $$\begin{aligned}{} & {} \text {volume}\left( c^{i}\right) \nonumber \\ {}{} & {} =\left\{ \begin{array}{lr} \prod \limits _{k=1}^{m}\left( z^{r e f}-f_{k}\left( x^{i}\right) \right) , &{} \qquad \forall k\left( z^{r e f}-f_{k}\left( x^{i}\right) >0\right) \\ 0, &{} \exists k\left( z^{\text {ref}}-f_{k}\left( x^{i}\right) \le 0\right) . \end{array}\right. \nonumber \\ \end{aligned}$$

Here, *m* is the number of objectives. The higher the *HV*, the better the convergence and distribution of the solutions.

**Fig. 7:**
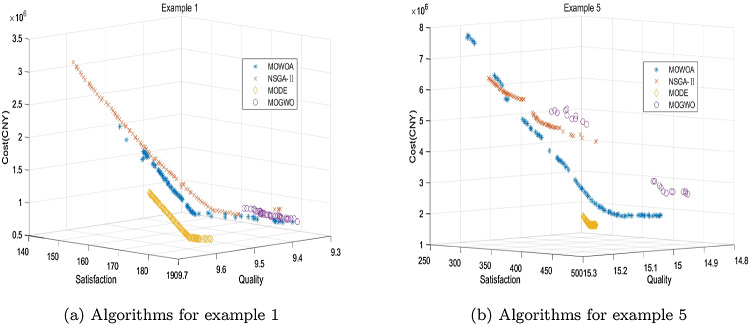
Comparison of Pareto frontiers between algorithms

Performance comparison of the four algorithms is shown in Table 7. The bold values indicate that the performance of this indicator is optimal among all the algorithms. The Pareto frontier comparison of the four algorithms is depicted in Fig. 7. The number of non-dominated tiers of NSGA-II, MODE, and MOWOA can all converge to 1 in 400 iterations. MOGWO is not convergent in all cases. From the perspective of solution diversity, MOWOA and MODE are better than NSGA-II and MOGWO. MOWOA can yield 100 different pareto solutions in all cases. There is only one case where different pareto solutions are less than 100 for MODE. From the perspective of computational efficiency, MOWOA is faster than the other three algorithms. From the perspective of HV, both the convergence and distribution of solutions of MOWOA are the best in all cases. Therefore, the improved MOWOA has the best performance among the four algorithms.

**Table 7:** Performance comparison between the four algorithms

Numerical example	Algorithm	Model size	Convergence iterations	Tiers	CPU time(s)	Different pareto solutions	HV($*10^7$)
1	NSGA-II	9$\times$5	222	1	150.188	97	0.661
	MODE		**213**	1	214.648	96	0.865
	MOGWO		–	2	162.960	23	–
	MOWOA		368	1	**131**.**198**	**100**	**3**.**910**
2	NSGA-II	10$\times$5	378	1	153.519	85	0.762
	MODE		**298**	1	220.621	100	0.620
	MOGWO		–	2	168.951	27	–
	MOWOA		369	1	**128**.**905**	**100**	**4**.**225**
3	NSGA-II	13$\times$6	355	1	205.447	88	10.361
	MODE		**180**	1	270.526	100	2.446
	MOGWO		–	2	231.141	20	–
	MOWOA		373	1	**139**.**534**	**100**	**16**.**880**
4	NSGA-II	16$\times$7	**275**	1	184.021	92	30.168
	MODE		386	1	336.022	100	47.305
	MOGWO		–	2	246.011	22	–
	MOWOA		317	1	**153**.**395**	**100**	**49**.**080**
5	NSGA-II	19$\times$8	**289**	1	238.611	84	17.583
	MODE		366	1	417.341	100	18.310
	MOGWO		–	2	298.865	23	–
	MOWOA		360	1	**188**.**120**	**100**	**47**.**695**
6	NSGA-II	22$\times$9	350	1	221.194	83	5.638
	MODE		396	1	463.810	100	47.008
	MOGWO		–	2	234.780	18	–
	MOWOA		**340**	1	**200**.**830**	**100**	**50**.**397**
7	NSGA-II	30$\times$10	377	1	279.631	90	8.737
	MODE		379	1	706.985	100	2.948
	MOGWO		–	2	312.982	15	–
	MOWOA		**316**	1	**214**.**351**	**100**	**26**.**727**
8	NSGA-II	40$\times$10	374	1	300.880	81	12.670
	MODE		389	1	724.026	100	76.330
	MOGWO		–	2	293.951	11	–
	MOWOA		**373**	1	**256**.**655**	**100**	**213**.**220**
9	NSGA-II	50$\times$10	385	1	336.682	86	3.691
	MODE		397	1	988.276	100	3.441
	MOGWO		–	2	288.461	15	–
	MOWOA		**308**	1	**239**.**865**	**100**	**35**.**668**

Model size- $$\left| I \right| \times \left| J \right| $$

**Table 8:** Comparison of the two decision-making methods

Solution no.	Method	Obj.1	Obj.2	Obj.3	Shortage
1	One-stage	9.601	144.247	3,124,246	24,923
	Two-stage	9.602	183.229	1,054,183	0
	Gap	$< 0.1\%$	27.02%	66.26%	100%
2	One-stage	9.545	170.367	1,591,669	7913
	Two-stage	9.626	170.433	1,859,612	7435
	Gap	0.85%	$< 0.1\%$	16.83%	6.04%
3	One-stage	9.562	164.150	1,925,736	11,491
	Two-stage	9.628	169.189	1,928,258	8131
	Gap	0.69%	3.07%	<$-$0.1%	29.24%

**Fig. 8:**
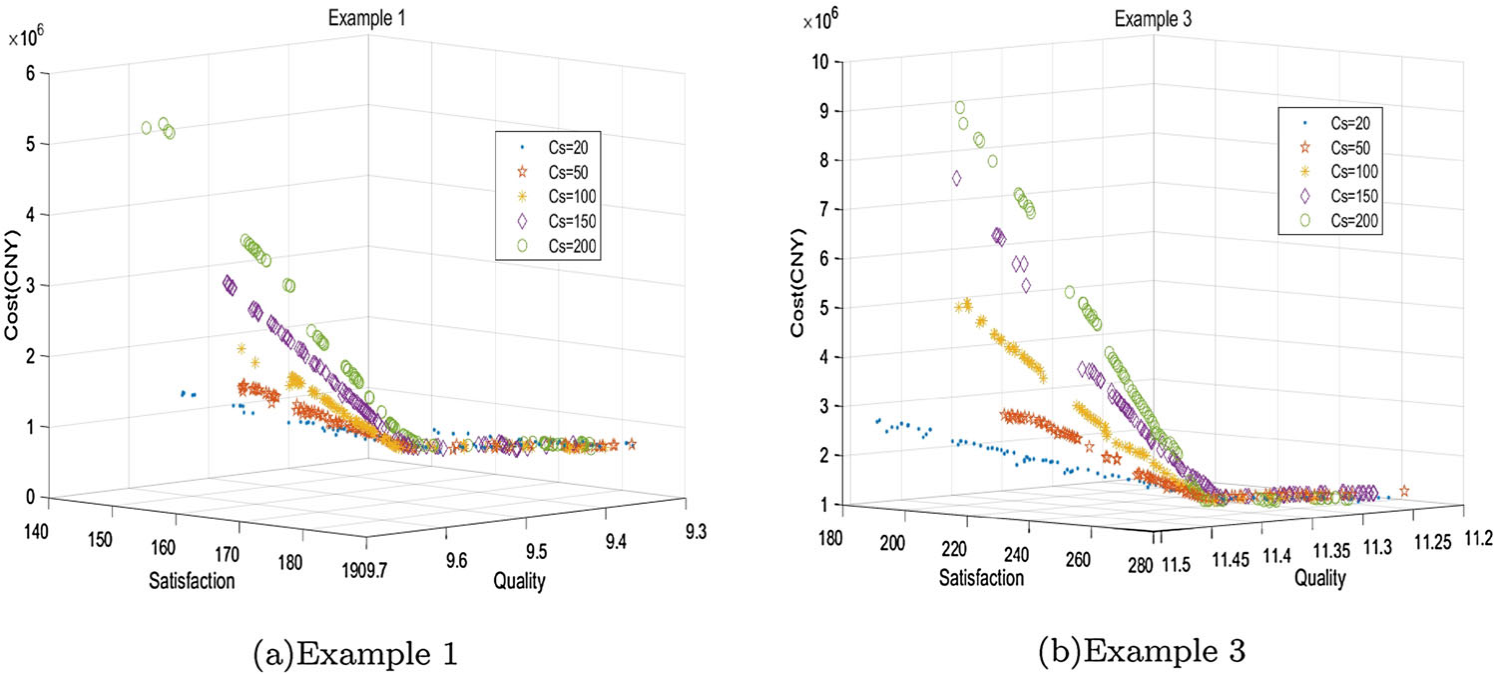
Pareto frontiers under different shortage penalty costs

**Fig. 9:**
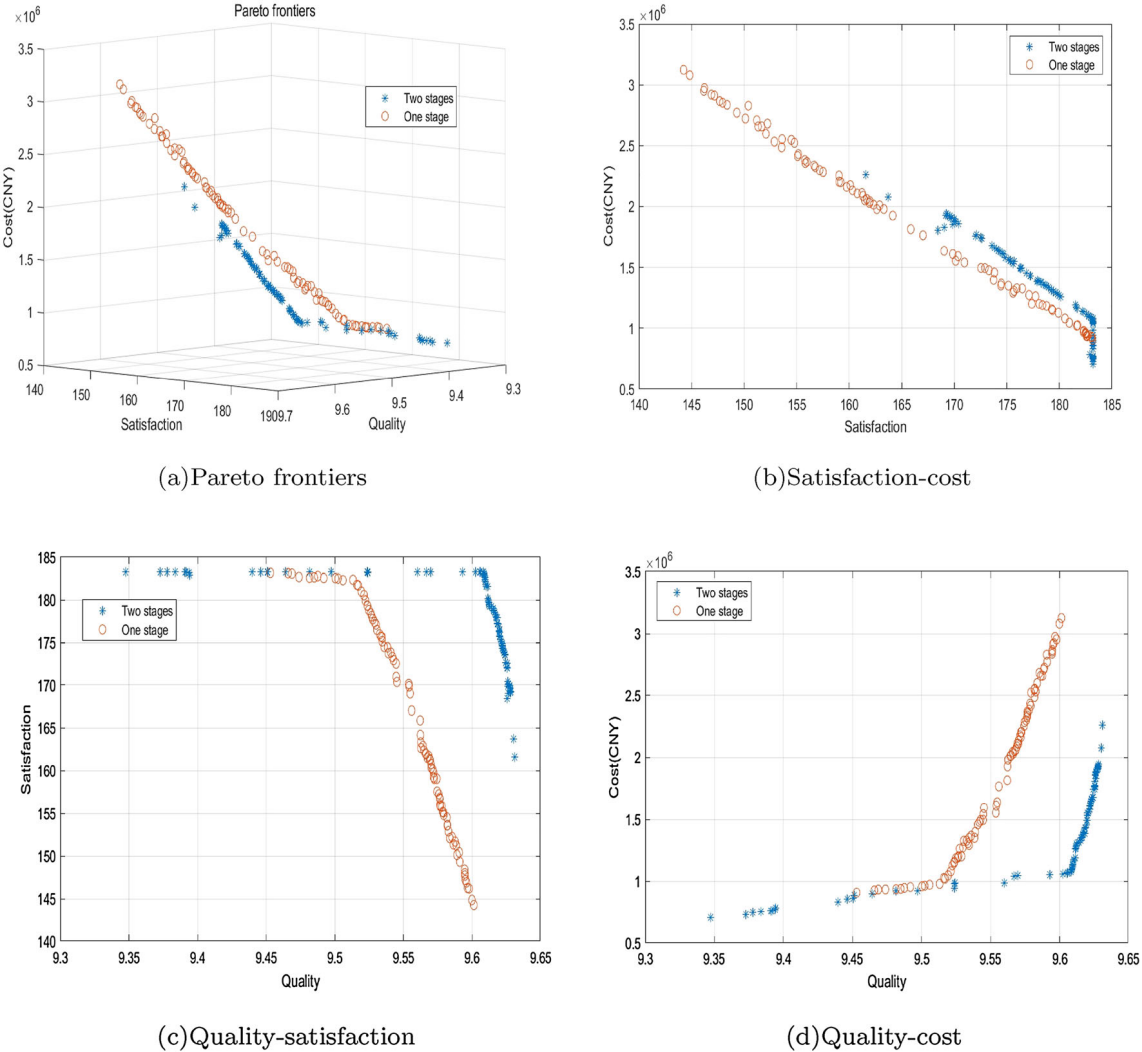
Schematic diagram of the comparison of the two decision-making methods

### Sensitivity analysis

To analyze the impact of blood shortage on the objectives of total cost, quality of transshipped blood, and satisfaction, sensitivity analysis is conducted with respect to the penalty cost ${{c}_{s}}$. Set ${{c}_{s}}$ as 20, 50, 100, 150, and 200 (CNY), respectively. Figure 8a shows the Pareto frontiers under different shortage penalty costs for Numerical Example 1. Figure 8b shows the sensitivity analysis for Numerical Example 3. The curves of the two figures have similar characteristics. With the increase of ${{c}_{s}}$, the total cost and satisfaction will also increase. ${{c}_{s}}$ has little influence on the quality of transshipped blood products.

### Two-stage decision-making versus one-stage decision-making

Figure 9 shows the comparison of the Pareto frontiers between two-stage decision-making and one-stage decision-making methods. From Fig. 9, it is seen that the two-stage decision-making is superior to one-stage decision-making on all objectives. To further compare the performance of the two decision-making methods, the solutions obtained by the two methods having a gap$\le \left| \pm 0.1\% \right|$ between different Pareto solutions for an objective are selected, and their optimization degrees are compared; see Table 8. For any one of the three objectives obtained by the one-stage and the two-stage decision-making methods, if the difference is within 0.1% of this objective, the two-stage decision-making is better than the one-stage decision-making with reference to the other two objectives and the shortage indicator. For example, in Solution No.1, the value of the quality objective is almost the same for the two methods, but the value of the quality objective increases by 27.02%, the total cost decreases by 66.26%, and the shortage cost decreases by 100%.

## Conclusions

This paper proposed a two-stage transshipment-allocation of blood products in the context of the COVID-19 epidemic. In the first stage, the inventory of affected banks can only be replenished from external blood banks. In the second stage, considering the reduced risk level of epidemic, the inventory of affected banks in low-risk areas can be replenished not only from external blood banks, but also partially from local blood collection. In view of this scenario, a multiple-objective optimization model was constructed to reflect the reasonable arrangement of transshipment blood from external blood banks by maximizing the quality of transshipment blood, and the satisfaction level of blood allocation to reflect the effectiveness of emergency blood allocation from blood banks to hospitals, and minimizing the system cost which contains the penalty cost for blood shortage to reduce the shortage.

Through the analysis of the proposed model, an improved integer-coded hybrid MOWOA with greedy search rules was proposed to solve the proposed multi-objective optimization problem. Numerical simulation shows that the performance of our proposed hybrid MOWOA is better than NSGA-II, MODE, and MOGWO. The numerical results also show that the two-stage decision-making is superior to one-stage decision-making on all objectives.

Future research can consider the compatibility of blood groups in the model and study the two-stage blood transshipment-allocation problem of blood group substitution.
